# Supplementary material for: Shared Treg dysregulation underlies psoriasis vulgaris and atopic dermatitis through the PHF19-PRC2 epigenetic axis
Source: Front Immunol. 2026 Aug 20;17:1932788. doi: 10.3389/fimmu.2026.1932788 (PMC13538097; doi:10.3389/fimmu.2026.1932788)
Supplement: Supplementary file 3 [file DataSheet3.zip › Additional files/Additional file 2/56.HLA-DRB5.forest.pdf]

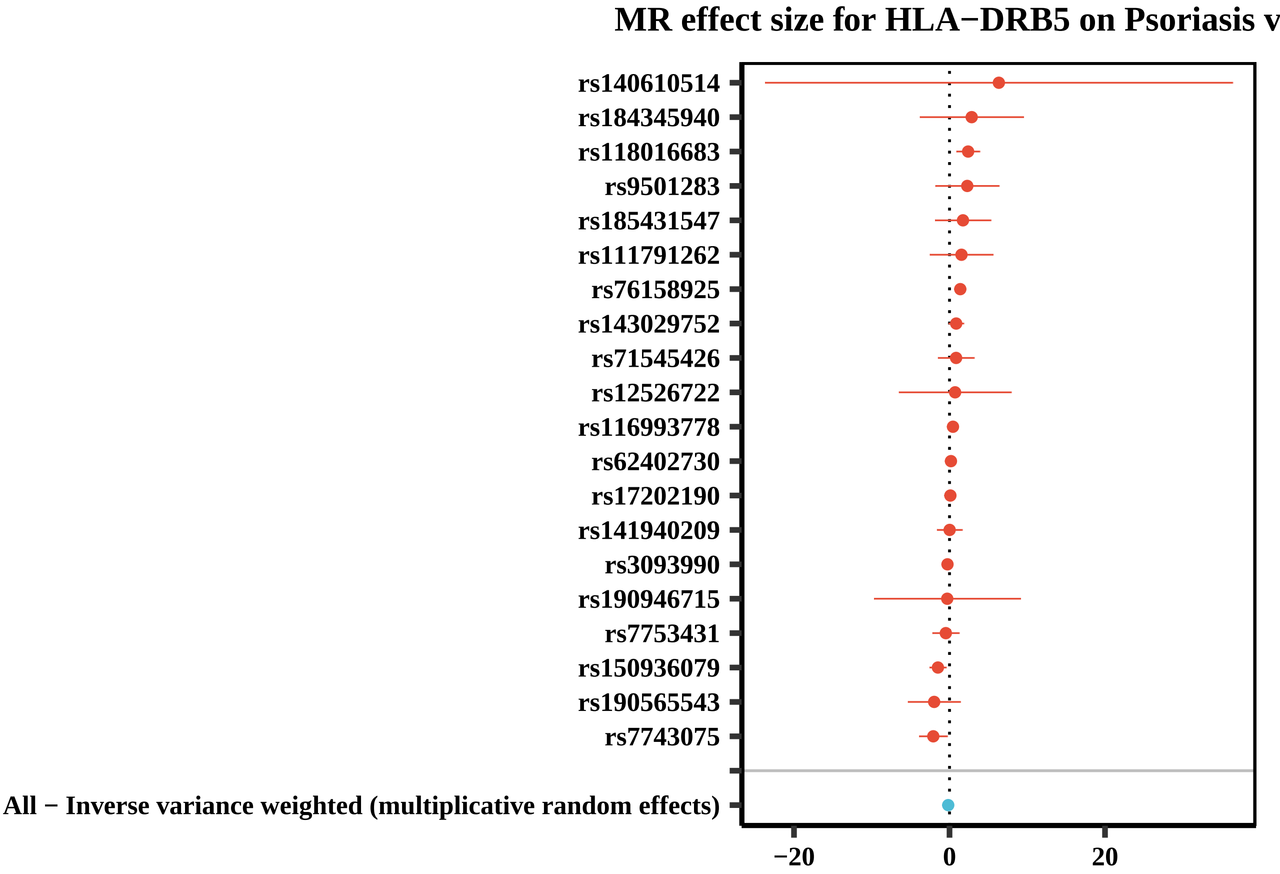
MR effect size for HLA−DRB5 on Psoriasis v
rs140610514
rs184345940
rs118016683
rs9501283
rs185431547
rs111791262
rs76158925
rs143029752
rs71545426
rs12526722
rs116993778
rs62402730
rs17202190
rs141940209
rs3093990
rs190946715
rs7753431
rs150936079
rs190565543
rs7743075
All − Inverse variance weighted (multiplicative random effects)
−20
0
20
